# Morphological constraints in hymenopteran forewings limit flight efficiency optimization

**DOI:** 10.1098/rsos.250224

**Published:** 2025-07-16

**Authors:** Iman Fadel, Pablo S. Milla Carmona, Yuming Liu, Emily J. Rayfield, Philip C. J. Donoghue

**Affiliations:** ^1^ School of Earth Sciences, University of Bristol, Bristol, UK

**Keywords:** performance landscapes, optimality, finite element analysis, elliptical Fourier analysis, morphometrics, Hymenoptera

## Abstract

The evolution of wings and flapping flight was integral to the radiation of Pterygota, but little is known about the factors underpinning the morphological disparity of insect wings. We use a theoretical morphospace approach to investigate forewing morphology across the four major clades in Hymenoptera (sawflies, wasps, bees and ants). Using elliptical Fourier analysis we quantified the outline of 298 forewings and generated 494 theoretical forms plotted within a morphospace. Theoretical forewing shapes were analysed across three metrics for flight performance that are antagonistic and ranked subsequently according to their functional optimization. The results show theoretical wings with larger, rounder apical tips were most optimized for a trade-off between reducing induced drag and increasing both lift production and breakage resistance. Empirical forewings cluster in a suboptimal region of theoretical morphospace exhibiting moderate flight performance. Phylomorphospace analysis reveals high levels of convergence in wing shapes across Hymenoptera, with a weak but significant phylogenetic signal. Regression analyses found significant allometric covariation but no significant relationship with environmental measures (temperature and precipitation) on forewing morphology. These findings demonstrate that hymenopteran wing morphologies are not optimized for flight function. Instead, function and allometry act in concert to constrain the variation of hymenopteran forewing morphologies.

## 1. Introduction

Hymenoptera, with suborders Apocrita (ants, bees, wasps) and Symphyta (sawflies, including woodwasps), comprise over 150 000 described species and are considered to surpass Coleoptera (beetles) as the most speciose insect order, given the vast array of parasitoid wasp species that are still undescribed [1,2]. Hymenoptera is not only hyperdiverse but also exhibits broad disparity across: morphology (including body size), habitats and biogeographic clines, and ecological roles [1,3]. Hymenoptera has earned widespread attention in conservation efforts and scientific study due to their ecological, agricultural and economic significance [4]. Hymenopteran flight kinematics and aerodynamic performance has been investigated through experimental and mathematical studies, with recent advancements from finite element analysis and computational fluid dynamic modelling [5–10]. Associated musculature and wing structure, particularly venation, have also been explored with reference to their effects on aerodynamic performance [7,11–13]. Notably, the majority of hymenopterans operate using asynchronous flight musculature allowing for increased wing beat frequency and therefore increased aerodynamic performance [14]. Compared with other pterygote orders, Hymenoptera display sparse venation patterns, yet the scaffolding nature of the networks, resilin joints and the flexible region on the forewing leading edge (‘costal break’) have all been shown to influence aerodynamic performance [9,15,16]. Most significantly, previous research has focussed on descriptions of hymenopteran wing morphology (and of insects generally) and their effects on flight function and adaptation, including the presence of a positive allometric covariation between wing and body size in hymenopteran groups [17]. This study aims to corroborate allometric covariations using an order-wide sampling of Hymenoptera. Wing size has also been linked to environmental and climatic factors such as temperature, precipitation, biogeography and species diversity [18–20]. In contrast, there has been limited exploration of the effect of habitat and environmental factors on wing shape across insects generally and Hymenoptera in particular [19,21,22]. Here, we explore the effects of environmental factors on hymenopteran forewing shape variation. Morphometric analyses on wing shape variation have been successfully employed in studies of taxonomic variation within Hymenoptera [23]. However, there is a notable gap in research into the evolutionary origins, drivers and constraints of wing morphologies which result in the disparity of wing forms we observe in nature [24–27].

We address this gap in examining the disparity and degree of flight function optimization in hymenopteran insect wings within a theoretical morphology approach. Theoretical morphology involves mathematical generation of an array of theoretical shapes to be visualized within a morphometric space (morphospace) [28]. While morphological disparity can be characterized through occupation of empirical shape space, a theoretical morphospace approach permits further characterization and functional analysis of morphologies in unoccupied territories of shape space [29–31]. This facilitates analysis of morphological variation as well as identification of optimal morphologies, whether or not they exist in nature, all unencumbered by prior assumptions of adaptive optimality [28]. Ultimately the theoretical morphology approach facilitates tests of causal evolutionary hypotheses of wing morphology within and beyond shapes realized in nature. With the aim of elucidating functional optimization across this broad spectrum of morphological variation, our functional analyses were performed using theoretical morphologies only and the results then compared against the biologically realized wing forms. Here, we characterize flight efficiency across theoretical wing planforms extrapolated from 298 extant forewing outlines as a trade-off between three flight metrics calculable from wing shapes: lift production, breakage resistance and reduction in induced drag. These flight functional metrics are antagonistic yet they have all been shown to correlate with wing shape in insects, with greater lift to drag ratios associated with increased radius of gyration and slender wing forms, and consequently an increased risk for wing breakage [6,8,17,24,32,33]. While a reduction in induced drag present in high aspect ratio wings is primarily associated with improved efficiency in patterns of gliding flight, the lift production associated with increased second moment of area is favourable for flapping flight particularly with additional wing loading [32,34,35]. Our study combines theoretical morphospace with a recently developed Pareto ranking algorithm, designed to consider performance rather than fitness, to investigate whether wing forms are optimized for flight function [36]. Pareto frameworks are often implemented in the evaluation of functional trade-offs and in determining optimal morphology; this is critical as individual traits are seldom associated with a singular function and frequently represent a phenotypic compromise [37]. Using the insect order Hymenoptera as a model system, we investigate the drivers and limitations of morphological evolution by exploiting the parallel evolution of traits in ecologically and phenotypically disparate sister clades of Formicidae (ant monophyly) and Apoidea (containing monophyletic bees and some wasps).

## 2. Material and methods

**Figure 1.**
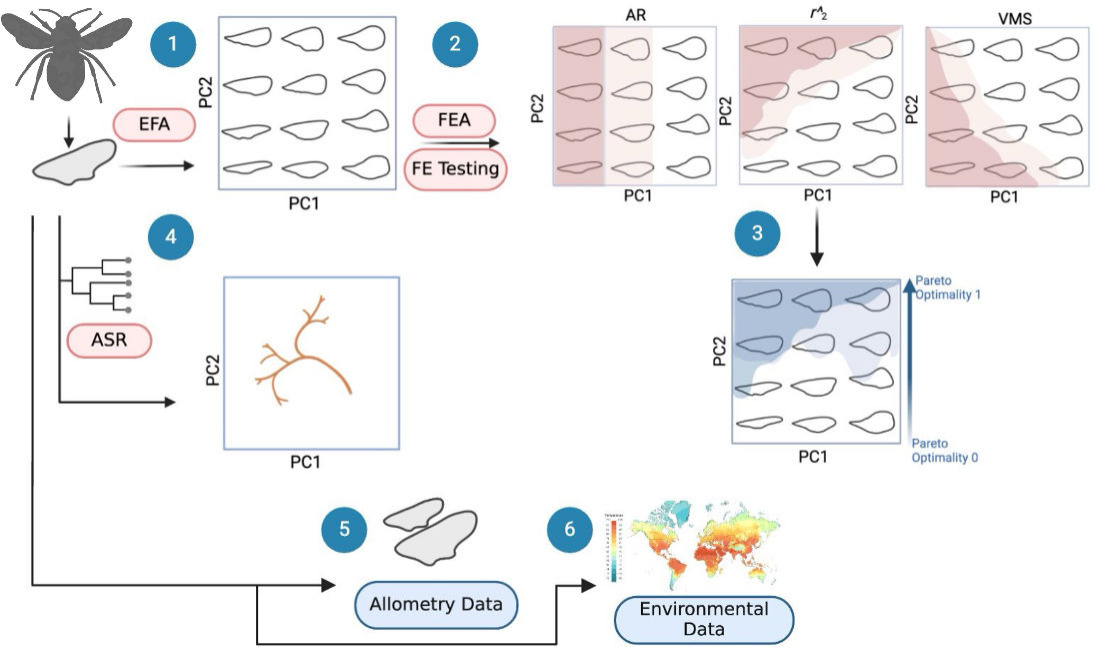
Methodological pipeline showing data collection and transformation of wing images into binary outlines to be characterized into quantifiable shape data using EFA (1). Extrapolation of EFA shape data to construct theoretical morphologies (visualized in a theoretical morphospace) for functional testing (2). The functional performance metrics were aspect ratio (AR), radius to the second moment of area ($\hat{r_{2}}$) and Von Mises stress (VMS). VMS was modelled using finite element analysis (FEA). Pareto ranking for optimality of the theoretical wing morphologies with reference to a trade-off between the three mentioned flight efficiency metrics (3). The EFA shape data were also used alongside a time-calibrated phylogeny and ancestral state reconstruction (ASR) to construct a phylomorphospace (4), size data to perform an allometric regression (5) and climate data (temperature and precipitation) for an environmental regression (6).

Our methodological pipeline (figure 1) follows the analysis of hymenopteran forewing outlines into quantifiable shape data using elliptical Fourier analysis (EFA) and simplified using principal component analysis (PCA) (1), which was then used to explore the impact of functional (2,3), evolutionary (4), developmental (5) and environmental (6) potential constraints on the evolution of wing shapes. Theoretical wing morphologies provided the basis for functional testing (2). This allowed for visual comparison between empirical morphospace occupation and the most optimal theoretical wing forms when considering a Pareto ranked trade-off between induced drag reduction (AR), lift production ($\hat{r_{\text{2}}}$) and breakage resistance (VMS) as combined metrics for flight efficiency. The influence of evolutionary history was explored using a phylomorphospace and assessed for phylogenetic signal (4). The impact of allometric developmental constraint (5) as well as the environmental effects of average temperature and precipitation (6) on wing shape variation were analysed using simple linear regressions.

### 2.1. Data collection and pre-analytical processing

We approximate hymenopteran forewing morphologies as two-dimensional single pixel outlines. Forewing images for 298 taxa were sourced from a variety of online museum collections and published literature, representing species across 66 hymenopteran families (electronic supplementary material, table S1). Each taxon was characterized according to the four major hymenopteran clades; monophyletic ants (Formicidae) and bees (Anthophila), paraphyletic wasps (all apocritans excluded from the previous two groupings), as well as sawflies (including woodwasps, paraphyletic suborder Symphyta) [1]. Alongside forewing images, associated size (wing length) and locality data (most dense occurrence geographic coordinates with mean annual temperature (MAT) and mean annual rainfall (MAR) were obtained, where possible, by cross-referencing locality data with the CHELSA climatology database [38,39]). This study focusses solely on forewings despite the coupled forewings and hindwings that occur in Hymenoptera. Previous ablation studies have shown that hymenopteran forewings are the primary drivers for flight ability and hindwings do not provide informative insight into flight function when considered separately [40]. While the total coupled forewing–hindwing unit would provide insight into flight efficiency, the combined wings are not applicable to this shape-focussed study as they are dependent on the inclusion of structural elements with discordant material properties to the wing membrane (e.g. hamuli hooks) [41].

### 2.2. Shape quantification and theoretical morphospace

Shape quantification and theoretical morphospace generation was performed using the MATLAB package ‘theofun’ [36]. Two-dimensional outlines were reconstructed and quantified using EFA, sequentially decomposing each shape into closed elliptical harmonics without requirement for homologous landmarking, and variation due to differences in size, rotation and position of the wing forms was standardized to isolate shape-derived variation [36,42–44]. Each wing outline was characterized by 150 discrete (*x*,*y*) coordinates yielding nine elliptical harmonics (validated to 99.5% harmonic power) [45,46]. Four Fourier coefficients were used to describe each of the nine harmonics, providing an empirical dataset that covered 33-character coefficients [36,45]. The shape variation described across these coefficients was summarized using PCA generating an empirical morphospace totalling 79% shape variation across the first two PC axes [36,46]. Four hundred and ninety four theoretical wing shapes were generated using the theofun package [36] through modified calculations across the empirical shape data and plotted equidistant across a 26 × 19 morphospace. The extended limits of this morphospace allowed us to explore the proportion of shape space defined by the empirical forewing variation with an additional border of shape space equivalent to 20% that of the empirical shape space. This allowed for visualization of theoretical forms at the limits of geometric possibility without unnecessarily considering regions of geometric impossibility [36]. Theoretical wing forms exhibiting points of self-intersection are greyed-out in the theoretical morphospace plots and they were excluded from functional analyses. Throughout this paper, all references to shape space, occupation and availability refer to this tested proportion of the infinite shape space and the abstraction to the first two principal components. Our morphospace fulfils the definition of a theoretical morphospace in all aspects except one [28,47,48]. Crucially, its dimensions are based on geometric models of form and encompass variation that extends beyond the bounds of variation in empirical data. However, our theoretical morphospace is derived from empirical data which McGhee [28] argues is not a characteristic of theoretical morphospaces [28]. However, as argued by Deakin *et al*. [36], this is a false expectation since even the seminal theoretical morphospace study of [49,50] was explicitly informed by his prior characterization of shell coiling parameters in molluscs [49–51]. As such, we follow Deakin *et al*. [36], Rawson *et al*. [31], Liu *et al*. [32] and Berks *et al*. [52] in arguing that our approach fulfils the expectations of a theoretical morphospace and it provides for a more flexible approach to characterizing complex morphology than simple generative models alone.

### 2.3. Functional analyses and pareto optimality ranking

Functional performance analyses were performed for three flight efficiency metrics across the 437 geometrically viable theoretical shapes. Performance landscapes were generated for each functional proxy. These landscapes are not synonymous with adaptive landscapes as representative of performance only and not fitness.

The flight efficiency metrics tested were (i) aspect ratio (AR), (ii) radius to the second moment of area ($\hat{r_{\text{2}}}$) and (iii) von Mises stress (VMS). These metrics were chosen because they can be calculated from two-dimensional wing outlines alone and thus are appropriate for analysis of theoretical wing shapes and suitable for functional testing as insect wings remain largely flat through flight strokes with minor cambering or twisting [53]. These metrics have previously been used as approximations for induced drag reduction, lift production and breakage resistance, respectively [6,7,24,54]. AR and $\hat{r_{\text{2}}}$ are non-dimensional metrics that are often used in studies of aerodynamic efficiency in insect wings [6,7,24,32,33]. Wing membranes are subject to bending and camber through flapping flight and therefore resistance to breakage or critical stress is also an important measure of aerodynamic performance [8,32].

AR is the primary quantifiable characteristic of wing shape, both in insect and vertebrate wings, and is an often used proxy for flight power efficiency [55]. Increased AR produces a reduction in the effect of wing tip vortices on flow detachment and consequently reduce induced drag, which in turn reduces the power expenditure thereby increasing aerodynamic efficiency [32,56]. Generally, AR is associated with slenderness of the wing, and as such longer slender wing forms are associated with improved flight efficiency [56]. The effect of AR in flapping flight is complex and previous reports have shown that with very high AR there is a break in the leading-edge vortex reducing the lift coefficient, however, it has also been shown that despite this break in flow, the lift/drag ratio continues to increase at high AR [55,56]. AR was calculated with reference to wingspan (*R*) and wing area (*S*) using the following formula [56]:


$$AR=\frac{R^{2}}{S}$$

Radius to the second moment of area ($\hat{r_{\text{2}}}$), often described as radius of gyration and characterized by a comparatively greater wing area in the distal portion, is used as a proxy for aerodynamic lift production in which a greater apical area linearly increases mean lift force in flapping flight in insects [6,7,24,34,57]. $\hat{r_{\text{2}}}$ is a non-dimensional shape metric, and was calculated as below, with reference to normalized chord (*c*) and radial distance from the wing base (d*r*) [7,24]:


$$\hat{r_{\text{2}}}=\int_{0}^{1}\hat{C}\hat{\;r^{\text{2}}}\;\hat{dr}.$$

This study follows previous research in the assumption that increased AR and $\hat{r_{\text{2}}}$ are preferential for flight efficiency [24,56,58].

VMS was used to simulate wing breakage resistance, by which a lower VMS indicates higher breakage resistance. Wing breakage resistance was modelled using a finite element analysis (FEA) pipeline as part of the theofun package [36]. Each theoretical wing form, normalized to equal thickness and surface area to correct for size variance, was discretized into a three-dimensional mesh (2500 triangular elements) and uniformly assigned a Young’s modulus of 1 GPa and a Poisson’s ratio of 0.49, consistent with previous research modelling insect wing membrane deformation as the theoretical shapes used do not account for internal venation structures [8,59]. Additionally, the theoretical planforms were constrained at the proximal wing base by four totally fixed nodes (no displacement or rotation) with a loading force of 0.003 N applied perpendicular to the apical wing tip consistent with flight camber [8,32,60]. The median value for von Mises stress for each FE model was then used to construct a performance landscape [31,32,36].

We use a Pareto optimality ranking method, as introduced in [36], with a basis in the Goldberg Pareto ranking system [31,32,36,52,61]. This method allowed us to combine two or more individual performance proxies into a single metric describing global performance, taking into consideration potential antagonistic relationships between them. This was used to sequentially score the 437 theoretical wing shapes with respect to the functional trade-off described between antagonistic functional proxies AR, $\hat{r_{\text{2}}}$ and VMS. All three functional metrics to be assigned equal weighting, which is beneficial in considering theoretical morphologies for which performance preferences would otherwise need estimation [36,52,61]. This system evaluates the set of solutions to the theoretical forms and defines a Pareto optimal subset [36,52,61] describing the solutions which exist at the ‘Pareto front’ of peak optimality at which point the performance of each metric cannot be maximized without detriment to another [36,52,61]. This provides a binary score of 1 for the Pareto optimal subset (or Pareto front) and 0 for sub-optimal solutions [36,52,61]. With the Pareto optimal subset defined we are now able to score solutions dependent on their proximity to the Pareto front. This uses the Pareto rank ratio which combines both the Goldberg ranking, for iteratively determining the Pareto optimal subset from a set of solutions and removing it, along with a reverse optimality ranking (observed as increased optimality with decreased performance) [32,36,52]. Considering both optimal ranking and suboptimal ranking accounts for bias as a result of heterogeneity in performance space occupation [32,52]. Pareto rank ratio values describe the solution’s position in proximity to the optima, with values ranging from 0 (the worst solution as it is exceeded by all other solutions) to 1 (the solution defining the optimal front) [52]. Pareto rankings were displayed as an optimality landscape with the functional metric trade-off projected through the *z*-axis. The optimality scale of 0−1 was visualized through the landscape using a contour plot of ten shades of blue, with white indicating lowest optimality (score of 0) and dark blue indicating peak optimality (score of 1). While the focus of the study considered a three-way trade-off between AR, $\hat{r_{\text{2}}}$ and VMS, paired trade-offs between the three flight metrics were also performed (electronic supplementary material, figure S1).

### 2.4. Phylomorphospace and phylogenetic signal

The influence of evolutionary history on hymenopteran wing shape variation was investigated both statistically, by testing for phylogenetic signal, and visually with a phylomorphospace. Both utilized a phylogenetic tree spanning Hymenoptera and amended from a published time calibrated phylogeny at a species level to provide broad coverage across the empirical sample taxa [1]. The tree represented 139 of the 298 taxa (covering 54 out of 66 families, and all superfamilies) present in the dataset [1]. The phylogeny used was initially constructed from molecular analyses of 134 transcriptomes spanning Hymenoptera [1]. While it has been noted that this study included relatively denser sampling across Apocrita than for Symphyta, this is widely considered a robust phylogeny that has been corroborated by subsequent phylogenomic studies [1,62,63].

For analytical robustness and simplicity, this study constructs a phylomorphospace through projection of independently estimated ancestral state shapes onto the morphospace defined by our trait data (i.e. the ‘phylomorphospace analysis’ outlined in [64]). The phylomorphospace was generated in R, using packages ‘ape’, ‘geomorph’ and ‘phytools’ through comparing harmonic data with the modified phylogenetic tree [65–68]. From this, the shape data for 139 taxa were used to reconstruct ancestral states under the Brownian motion (BM) model of evolution without assumption of functional selection. The phylomorphospace was then visualized across the first two principal components along with the data points for the 159 taxa not represented in the phylogeny.

The phylogenetic signal present across 139 taxa representative of our dataset was calculated using a multivariate *K*-statistical test (*K*_mult_) across 9999 iterations [69]. The kappa statistic provides a ratio for the multivariate shape data for observed variation by the variation expected under BM (scored as 1) [64].

### 2.5. Allometric and environmental regression analyses

The developmental and environmental effects on forewing evolution in Hymenoptera were analysed through both simple linear regression and phylogenetic generalized least squares (PGLS) as follows.

A linear regression analysis was performed in R (packages ‘ape’, ‘Momocs’, ‘geomorph’ and ‘morphospace’) to ascertain the significance of the relationship between wing shape and wing size [65–67,70,71]. Fourier coefficients for PC1 were analysed against calculated centroid size for 225 taxa in the dataset. PC1 was chosen as this covers most shape variation (67%). Centroid size, as a proxy for wing size (and body size considering generalized co-scaling), was calculated using distances between the wing centroid and outline coordinates [72].

Climate data were collected for 264 of the taxa in the dataset, preferentially as geographic coordinates corresponding to densest occurrence, (based on the most recent records from the GBIF database) or directly from the original image sources, where available. Latitudinal and longitudinal coordinates were input into a climate database [38,39] to obtain mean annual temperature (MAT; °C) and mean annual rainfall (MAR; mm) for specific localities [38,73]. Temperature and precipitation data were independently regressed against the harmonic wing shape data from PC1 (as approx. 70% shape variation is captured in this axis). MAR was also regressed against PC2 shape data as higher precipitation levels are associated with decreased foraging and pollination (associated with increased radius of gyration) [74–76]. All environmental analyses were performed in R using packages ‘ape’ and ‘geomorph’ [65–67].

PGLS analyses were performed for each of the testing scenarios above (pertaining to size and environment) to consider phylogenetic non-independence within the dataset. All PGLS analyses were performed in R using ‘caper’ [77] along with the aforementioned packages. The PGLS analyses were performed with reference to a phylogenetic tree covering 139 taxa present in the dataset (as described above). This resulted in a loss of data resolution for the PGLS analyses compared with the simple linear regressions, with 73 of the 298 taxa accounting for size data and presence in the time-calibrated phylogeny, 67 of which also had recorded climate data. As such the results of the PGLS analyses were considered alongside and as supplementary to the simple linear regressions.

## 3. Results

### 3.1. Theoretical morphospace

**Figure 2.**
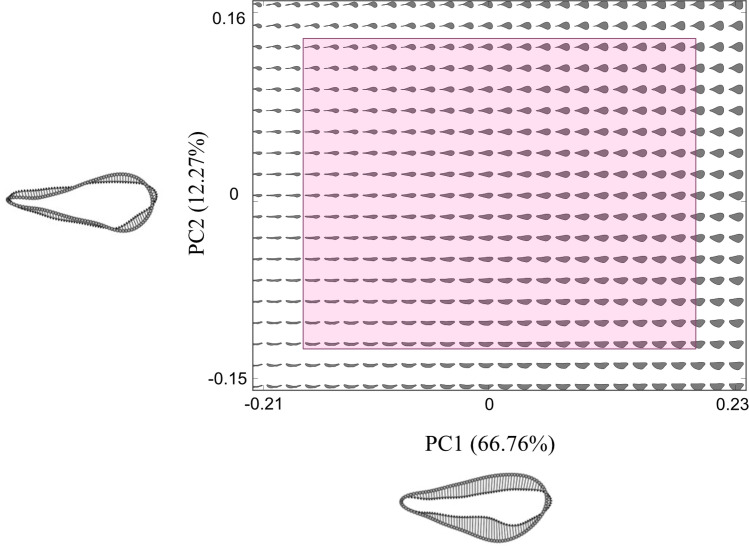
Theoretical morphospace depicting 494 theoretical hymenopteran wing shapes equally spaced in a 26 x 19 grid across a PC1/PC2 morphospace. All theoretical wings were standardized to a constant size, depicted size variation is an artefact of Matlab figure generation. The pink box highlights the area of shape space captured by the empirical dataset and is surrounded by a 20% border for further shape visualization. PC1 (*x*-axis) captures 66.76% and characterizes shape changes across the breadth of the wing planform from thin to broad with increasing values. PC2 (*y*-axis) captures 12.27% shape variation, characterized by an increase in size of the distal apical tip relative to the humeral wing base with increasing values (shape changes illustrated for each axis).

**Figure 3.**
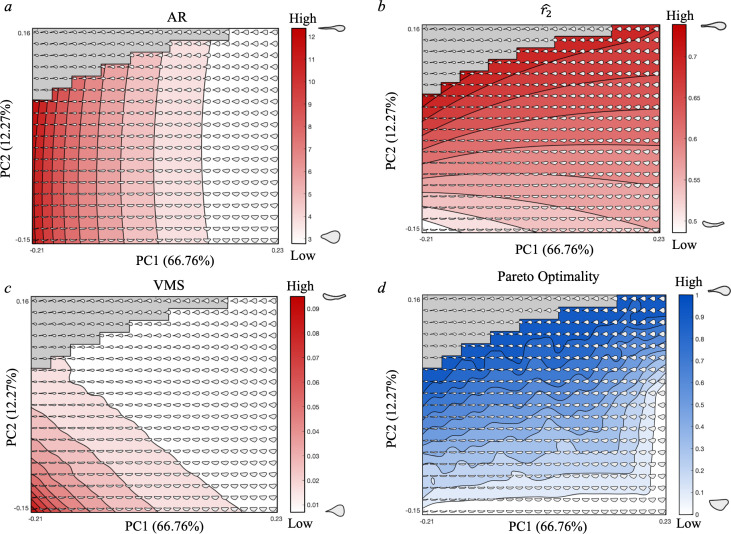
Performance landscapes (*a–c*) for functional testing for flight efficiency metrics (*a*) aspect ratio (AR), (*b*) radius to the second moment of area ($\hat{r_{2}}$), (*c*) Von Mises stress (VMS), and optimality landscape (*d*) for Pareto optimality rankings. All four landscapes display 10 contours with shading corresponding to the scoring for performance or optimality as indicated by the heat-map scale bar, with a reference example of high and low scoring theoretical hymenopteran wing forms. Performance and optimality landscapes are overlayed atop the theoretical morphospace to visualize morphologies occupying each contour. The grey box in the top left corner indicates a region of impossible shape space and the 57 wing shapes excluded from functional testing. All theoretical wings were standardized to a constant size, depicted size variation is an artefact of Matlab figure generation.

Principal component analysis of the outline shape data captures 79% of shape variation (79%) on the first two axes of the theoretical morphospace (figure 2). PC1 (66.76% variation) describes shape changes from narrow to broader wing heights, while PC2 (12.27% variation) corresponds to increasing roundness and size of the apical wing tip relative to the humeral base. Four hundred and ninety-four theoretical morphologies populate the morphospace in a 26 × 19 equidistant grid capturing the breadth of geometric possibility of empirical shape space and the bordering 20%. 11.5% of the theoretical wing forms fall into an area of morphospace in the top left corner (intersection of low positive to negative values of PC1, with positive values of PC2) of the plot (figure 3*a*–*c*) where wings generally have greater length relative to width and a larger disparity between the size of the apical wing tip and humeral wing base. In this region, the shape extrapolation method has resulted in shapes that are self-intersecting. Such shapes are morphologically nonviable and were therefore omitted from functional testing.

### 3.2. Functional analyses

The performance landscape for AR (figure 3*a*) illustrates near vertical contour stripes, indicating AR is almost entirely influenced by PC1 scoring with little impact from PC2. The highest scoring wing forms (AR ≥12) occupy a narrow field of the morphospace at the lowest PC1 values and extending across PC2 from −0.5 to the boundary with impossible space (PC2 = 0.06). These wings are characterized by their slenderness (low width : height) and according to our functional hypotheses, are shapes associated with reduced drag. As the AR values decrease, the theoretical wing forms become broader. The poorest performing shapes (AR ≤3) occupy a considerably larger area of shape space from 0.1 to 0.23 on PC1.

Similarly, the performance landscape for $\hat{r_{\text{2}}}$ (our trait associated with aerodynamic lift production) shows the theoretical wing forms with the highest performance ($\hat{r_{\text{2}}}$ ≥ 0.74) occupying the smallest area of shape space (figure 3*b*). These forms, with relatively narrow humeral base but broader and rounder apical wing tips, are situated towards the top left of the morphospace adjacent to the impossible space. The poorest performing wing forms ($\hat{r_{\text{2}}}$ ≤0.51) are located to the lower-leftmost corner of the plot, extending further across PC1 (−0.22 to −0.15) than PC2 (−0.155 to −0.14). The general trend for $\hat{r_{\text{2}}}$ (as shown by the near horizontal contour lines) is horizontal, indicating a strong influence from PC2. The performance landscape is steeper at low PC1 values with contours distributed more tightly across the vertical. The landscape contours fan out as PC1 increases, indicating a reducing influence of PC1 on $\hat{r_{\text{2}}}$ as the wing forms increase in breadth.

The VMS performance landscape (figure 3*c*) differs from the previous two metrics, in that the relationship between VMS values and breakage resistance is inversely proportional. This means that richer colours represent higher stress and by inference wing planforms that are more likely to break and therefore with less optimal performance. The theoretical shapes were scored by the mean VMS experienced under assigned FEA boundary conditions. The landscape shows theoretical forms corresponding to highest performance (VMS ≤ 0.01) occupying the largest area of shape space, although still limited by impossible shape space, with decreasing contour area as mean VMS increases. The poorer performing shapes (VMS ≥0.05) are narrow across the wing form with little definition between the base and wing tip. These shapes occupy a small portion of total shape space with similar influence from both axes (PC1 range from −0.22 to −0.15, PC2 range from −0.16 to −0.1).

### 3.3. Pareto optimality

The flight efficiency trade-off between the three tested functional metrics (AR, $\hat{r_{\text{2}}}$ and VMS) was quantitatively ranked using a Pareto approach [36] and visualized as an optimality landscape (figure 3*d*). The ten contour plot ranges from most optimal theoretical wing shapes for the trade-off at the ‘Pareto front’ (score of 1) to the least optimal shapes (score of 0). The landscape shows a near vertical progression (with gentle incline in optimality as PC1 increases) from most optimal wing shapes (high PC2 values) to least optimal shapes (low PC2 values). There is a shift towards a more diagonal trend as the landscape steepens around PC1 = 0.1, indicative of a lower optimality at PC1 values > 0.1. The Pareto front borders the impossible shape space, with high optimality shapes described as wing forms with as narrow a base and broad a tip as geometrically possible, and with a slight preference towards a more slender planform. Similarly, the least optimal shapes can be described as wings with similar breadth of wing base and wing tip (low PC2) as well as shapes which are most broad in height (high PC1). Comparisons between pairwise trade-offs for the three flight metrics showed near identical landscape patterning between the three-way trade-off and the pairwise trade-off between AR and VMS. The trade-off between AR and $\hat{r_{\text{2}}}$ also shows a similar pattern. The pairwise trade-off between VMS and $\hat{r_{\text{2}}}$ shows a pattern of increasing optimality towards the top right of the plot, rather than the top left as for all other combinations of traits (electronic supplementary material, figure S1).

### 3.4. Empirical occupation of theoretical morphospace

**Figure 4.**
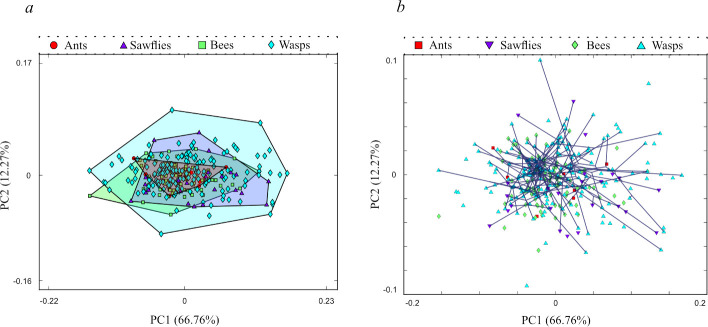
(*a*) Morphospace occupation of Hymenoptera, showing positions of taxa and convex hulls for each of the four major clades; ants (red circles), bees (green square), wasps (blue diamonds), and sawflies (purple triangles). (*b*) Phylomorphospace visualizing phylogenetic relationships between reconstructed ancestral forms and empirical taxa data points within theoretical shape space. Ants: red squares; bees: green diamonds; wasps: blue triangles; sawflies: purple inverted triangles. The data points for which no branches are connected represent the taxa in the dataset that do not appear in the phylogeny.

The empirical PC1–PC2 morphospace (figure 4*a*) shows the shape disparity of the 298 empirical taxa, categorized into the four major clades of Hymenoptera; ants (7), bees (95), wasps (172) and sawflies (including woodwasps, 24). There is significant overlap of the four convex hulls which capture the total observed shape variation for each clade. The majority of the data points across all clades are clustered centrally. Of the four clades, the wasps capture the most shape variation, and encompass the total variation for ants, sawflies, and all bee taxa (excluding 1, PC1 = −0.15 PC2 = −0.03). The taxa for all clades show a greater morphological disparity across PC1 (−0.15 −0.17) than PC2 (−0.09 −0.1), with certain wasp and sawfly taxa extending furthest across PC2. The empirical shape variation covers 24% of the morphospace (ants 2.6%, bees 9.6%, wasps 24%, sawflies 10%), leaving a considerable area of shape space unoccupied.

**Figure 5.**
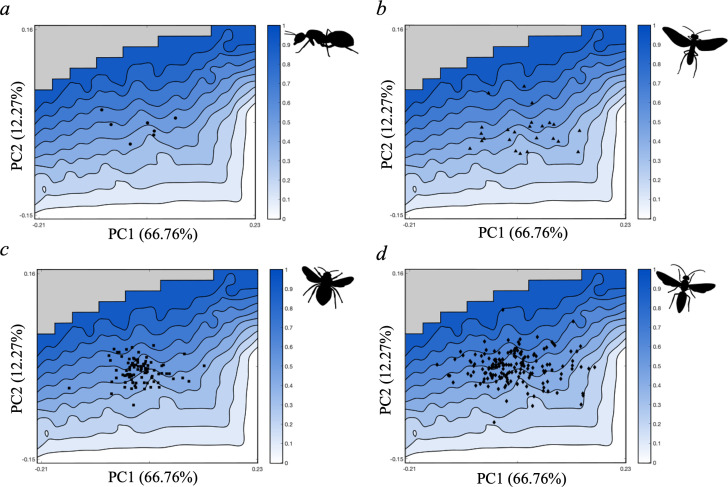
Pareto optimality landscape displaying the empirical distribution of hymenopteran forewings. Optimality of theoretical wing forms presented as a trade-off between flight efficiency metrics AR, ($\hat{r_{2}}$) and VMS ranging from optimal (1, dark blue) to poorly optimized (0, white). Each panel presents the optimality landscape superimposed with empirical data points for ant (*a*), sawfly (*b*), bee (*c*) and wasp (*d*) taxa. The grey box in the top left corner indicates a region of impossible shape space and the 57 wing shapes excluded from functional testing.

With the empirical data points superimposed over the Pareto optimality landscape, it is clear that the majority of the taxa in the dataset share shape space with theoretical forms scoring between 0.4 and 0.7 for flight efficiency optimality (figure 5). Ants (figure 5*a*) and bees (figure 5*c*) are particularly confined to this area of the performance landscape, however, sawflies (figure 5*b*) and wasps (figure 5*d*) exhibit greater variation in optimality. Sawflies cluster more densely in sub-optimal regions of shape space (<0.5), notably lower than the densest occupation of the other three groups, yet they also contain two of the higher performing taxa in the dataset (scoring approx. 0.8) (figure 5*c*). Wasps share shape space with theoretical forms spanning optimality scores 0.2−0.9 and contain the most optimal taxon (scoring ~0.9 and bordering the Pareto front) (figure 5*d*). The distribution of wasps in the lower right quadrant of the morphospace follow the optimality contours (score of 0.4) as the landscape steepens with increasing PC1 values (figure 5*d*). These patterns are largely similar for the pairwise trait comparisons.

### 3.5. Phylogenetic analyses and phylomorphospace

The phylomorphospace displays convergence through low degree of branch organization and significant overlapping centrally to the morphospace (figure 4*b*). This is indicative of many hymenopterans evolving similar wing forms independently, yet these have not evolved to occupy the regions of peak flight efficiency optimality (figure 5). The empirical data points show no discernible pattern of clade separation. The distribution of sawflies appears more widespread across the phylomorphospace than do the three other clades. Conversely, bees demonstrate more contained clustering. A multivariate *K* statistical analysis indicates significantly weaker phylogenetic signal than would be expected under a BM model (*K* = 0.46345, *p* = 1 × 10^–4^).

### 3.6. Allometric and environmental analyses

The allometric regression analysis of 225 empirical taxa showed significant covariation between wing size (log centroid size) and wing shape (using PC1 shape coefficients), as shown in (table 1). While the analysis indicated a significant relationship, the *R*^2^ value demonstrates that wing size only accounts for a small percentage (approx. 10%) of the morphological variation across PC1. The negative trend observed indicates decreasing wing size as wing forms broaden. A PGLS analysis for covariation between wing centroid size and wing shape, using 73 empirical taxa, indicated a slight negative correlation with no statistical significance (table 2).

**Table 1.** Results from simple regression analyses to test for covarying relationships between wing morphology and developmental or environmental explanatory variables (for each test the explanatory variable is listed first, and the dependent variable second). Significant results are highlighted in bold and with an asterisk.

test	multiple *R*^2^	adjusted *R*^2^	d.f.	*F*	*p*	slope
wing centroid size, wing shape	0.1079	0.1039	1, 223	26.97	*4.644 × 10^–7^	−0.02625
temperature, wing size	0.03664	0.03155	1, 189	7.189	*0.007983	0.6124
temperature, wing shape	0.01375	0.009971	1, 261	3.639	0.05755	1.253 × 10^–4^
rainfall, wing shape	0.002263	−0.001560	1, 261	0.5919	0.4424	8.176 × 10^–7^
rainfall, PC2	0.001548	−0.002277	1, 261	0.4048	0.5252	−3.414 × 10^–7^

**Table 2.** Results from PGLS analyses to test for covarying relationships between wing morphology and developmental or environmental explanatory variables without assumption of phylogenetic independence (for each test the explanatory variable is listed first, and the dependent variable second).

test	multiple *R*^2^	adjusted *R*^2^	d.f.	*F*	*p*	slope
wing centroid size, wing shape	0.001639	−0.01242	1, 71	0.1165	0.7338	−0.004159
temperature, wing size	0.01893	0.003839	1, 65	1.254	0.2668	0.008493
temperature, wing shape	0.0009510	−0.01442	1, 65	0.06187	0.05755	0.0002007
rainfall, wing shape	0.002394	−0.01295	1, 65	0.1560	0.6942	−5.089 × 10^–6^
rainfall, PC2	0.002081	−0.01327	1, 65	0.1355	0.7140	−2.100 × 10^–6^

When centroid size is analysed against MAT for 191 taxa a slight positive trend can be observed (slope 0.6124). This demonstrates an increase in wing size as MAT increases, however, the relationship only accounts for approximately 3% of wing size variation (table 1). The effect of temperature on wing shape also shows a very slight positive trend (slope value 1.253 × 10^–4^) yet the linear regression analysis found no statistical significance for covariation between wing shape and MAT. PGLS analysis of 67 empirical taxa found no significant relationship between mean annual temperature and either size or shape of wings (table 2).

A similar investigation into the relationship between MAR and wing shape shows a very slight positive trend (slope value 8.176 × 10^−7^) but no significant covariation (table 1). The results of the PGLS performed on 67 of the empirical taxa found again no significance but across a very slight negative trend (slope value of −5.0890 × 10^−6^) (table 2). Both the regression analysis between MAR and PC2 coefficients and the PGLS analysis also showed no statistical significance across a slight negative covariation trend (tables 1 and 2).

## 4. Discussion

The results of our phylomorphospace analysis show that morphological variation in hymenopteran forewings exhibits high levels of convergence. However, the empirical variation does not generally map well to theoretical wing morphologies at peak performance for the individual flight functions considered, nor optimally for the examined trade-off between these functional metrics. The following sections consider the causal bases for morphological evolution in hymenopteran forewings.

### 4.1. Convergent occupation of theoretical morphospace

Our sample of 298 hymenopteran forewings cluster centrally within the morphospace and with a high degree of overlapping shape variation across the four major clades (figure 4*a*). Combined with the results of the phylogenetic analyses, the minimal organization of phylomorphospace branches show a high level of convergent evolution of wing forms, with the phylogenetic signal found to be significant but weak (figure 4*b*). This convergent occupation sees the empirical forms spanning just under a quarter of the presented shape space, with a larger proportion of this morphospace unexplored in nature. It could be presumed that a portion of the unoccupied area may be a result of insufficient time for evolution to realize certain wing forms [30]. While this may be a contributing factor, macroevolutionary studies indicate that the rate of expansion of occupied morphospace typically decreases over time [78]. Of the four major clades, sawflies occupy the widest range of shape space relative to the number of taxa included in the dataset (wasps 172, bees 95, sawflies 24, ants 7). This supports the reported decoupling of taxonomic diversity and morphological disparity with Symphyta which is at an equal taxonomic level to Apocrita following divergence in the Permian [1,79,80]. Despite their earlier divergence, sawfly variation convergently occupies similar morphologies to those of the more derived hymenopteran groups; notably, their shape space is encompassed within that of the paraphyletic wasps. A similar observation can be made from comparison of ants (Formicidae) with bees (Anthophila). Formicidae resolves as a sister to Apoidea (clade containing bees and Apoid wasps) with divergence in the Jurassic, yet crown-group ants and bees are both estimated to have likely originated in the Early Cretaceous [1,81]. Despite their similar origination timings, bees have a far greater species diversity compared with the ants, yet still occupy the smallest area of our morphospace proportional to the number of taxa per group [82]. The increased degree of morphological clustering in bees has been previously documented with studies into numerous cryptic species in major bee families [83]. The morphological disparity for the ant group (Formicidae) is almost entirely contained by the bee and wasp shape variation and shares considerable overlap with the sawfly group (figure 4*a*). This is notable as the acquired wingless nature of ants sees winged forms primarily during reproduction [11]. Yet, the wing forms, which are shown to re-evolve with consistent morphologies after instances of winglessness [84], occupy similar wing shapes and thus landscape area as perennially flying taxa of bee, wasp, and sawfly groups.

Within the 76% of unoccupied morphospace, there is a region of impossible shape space (11% of total morphospace) containing biologically nonviable theoretical shapes, where the humeral wing base is so narrow relative to the apical wing tip that the extrusion method creates a self-intersection. These impossible wing forms border the areas of peak performance for all tested functional metrics and the Pareto front (figure 3). As these impossible forms are structurally inviable, it is reasonable that there may be further structural or ontogenetic limitations prohibiting realization of theoretical wing forms in the surrounding shape space despite their high functional optimization. Ontogenetic variation has been shown to alter the degree of morphospace occupation in previous studies and to act alongside allometry in the limiting of morphological variation [85].

The theoretical wings tested lack historicity and the theoretical morphospace provides a means of identifying functionally optimal shapes beyond those realized in nature. However, this requires the simplification of wings as isolated structures and without consideration of the effects body mass, flight musculature, and wing loading have on power requirements or wing deformation [13,54,86]. Body size is one of the principal developmental factors shown to drive shape disparity; the allometric effect of body size on wing shape in hymenopterans has been studied since the 1980s, often involving geometric morphometrics based on venation patterning [13,17]. Previous studies report an increase in aspect ratio correlated with an increase in body size, as we find in this study (using wing centroid size as a proxy) [13,17]. This has important biological significance as increasing aspect ratio decreases the drag coefficient and the power requirements on larger species for more efficient flight, which is offset in smaller species through increased wing beat frequency [24,56]. Our results from a simple linear regression corroborate a significant relationship between wing size and wing shape, with the allometric covariation corresponding to ~10% of morphological disparity (table 1). This is a small percentage but it explains morphological variation to a higher degree than the results of the phylogenetic analysis. The simplest interpretation of these results is that allometry plays a role in co-constraining hymenopteran wing form evolution, however it is worth noting that the observed allometric covariation may itself be a product of selection towards functional optima [87]. When considered alongside the results of the PGLS, we find no significant allometric covariation independent of phylogenetic relatedness (table 2). However, given the limitations of sample size for the analysis and the complementary results presented in the wider literature it is likely that significant effect of developmental constraints—whether or not entirely independent of evolutionary history—co-act alongside function in the evolution of wing morphology [13,17].

The effect of increasing global temperatures on wing size (and to a lesser extent wing shape) has been recently explored with growing concerns surrounding the climate crisis [19]. Our findings show no significant covariation between mean annual temperature and wing shape but do support a slight positive correlation between MAT and wing centroid size (table 1). While our findings agree with Allen’s rule suggesting reduced wing sizes in cooler climates, they contradict previous reports in Hymenoptera of a reduction in wing size with increasing MAT and, as such, contradict Bergmann’s rule (increasing body sizes as temperatures decrease) [19,72]. Our results are consistent with a previous study that indicates certain bee species may not follow Bergmann clines (presenting neutral or negative trends), likely the result of improved endothermy in eusocial species [88]. We similarly find no significant relationship between wing shape and mean annual precipitation which has previously been shown to prohibit foraging activity and influence flight activity in pollinating species [74–76].

### 4.2. Flight function and optimality

We find the empirical taxa included within the dataset do not share morphology with theoretical wing forms occupying regions of peak performance for AR, $\hat{r_{\text{2}}}$ or VMS (figure 3*a*–*c*). Peak performance of AR and $\hat{r_{\text{2}}}$ were assigned to the highest value for each metric. This is typical for flight studies, despite wide acknowledgement that high values of AR and $\hat{r_{\text{2}}}$ may lead to reduced manoeuvrability [24]. Manoeuvrability is undoubtedly important in functional flight efficiency trade-offs for Hymenoptera (e.g. pollinating species require the ability to trace movement of flowers [86]). However, this metric is not easily calculable from a wing planform alone and requires knowledge of both the angle and centre of mass of a body [7]. AR is generally considered the most important morphological metric for flight ability in insects and is the focus of many micro-air vehicle designs [56]. The performance landscape depicts theoretical wing forms with AR = 12 to be at peak performance (figure 3*a*). Previous research has shown a general trend for increase in lift : drag with increasing AR, however the trend begins to stabilize at AR = 3 and plateau at AR > 7 [55]. As all of the taxa in the dataset fall between 3 ≤ AR ≥ 8, we can determine that they operate at a high lift : drag ratio, and therefore, high performance despite not sharing morphospace occupation with theoretical shapes at AR values equal to or around 12. The taxon furthest from peak AR (figure 3*a*) belongs to Eucharitidae (a family of ant-parasitoid wasps); these wasps are small (<5 mm body length) and have highly specialized roles as parasitoids subsequently providing biocontrol of invasive ant species [89,90]. Insect miniaturization is particularly associated with both Coleoptera and Hymenoptera (primarily across parasitoid wasps) with previous research demonstrating a pattern of decreased AR and increased rounding of the apical wing tip in these smaller species [91]. Theoretical wing forms scoring highly for VMS (more prone to breakage) were those narrow across the planform or with extremely narrow humeral bases (figure 3*c*). This is expected as insect wing membranes are subject to high stress and deformation through flapping flight and concentrate flexural stiffness to the humeral base of wings [8]. Although previous studies highlight wing venation as significant in structural support and breakage resistance, hymenopterans have relatively sparse venation networks and so focus on wing shape alone should reflect breakage resistance [16,41,54]. Furthermore, in Hymenoptera, the stiffness of the wing base and the flexibility of the apical tip are shown to unidirectionally focus bending with camber thus aiding the wing to withstand further deformation through flapping flight [5]. While this study pertains to forewing morphology, as drivers of hymenopteran flight kinematics, the autapomorphic hamuli ‘hooks’ coupling fore and hind wings in Hymenoptera are shown to add elevated resistance to bending and deformation through flight [40]. Theoretical wing forms that are slender across the planform are similarly poorly performing with reference to $\hat{r_{\text{2}}}$ and do not occur in Hymenoptera (figure 3*b*). This is expected as flight requires sufficient lift production to support body weight even without additional prey or foraging load, and although flight musculature contributes to lift production, the wing properties hold considerable impact [3]. Pollinating hymenopterans are often subject to additional load bearing up to 150 times their body mass, in turn associated with maximized radius of gyration (PC2) [7,15].

The Pareto optimality landscapes for the paired trade-off between AR and VMS is near identical to the three-way trade-off landscape; this indicates that these two performance measures impart a greater influence towards the functional constraint of wing morphologies (as observed from the three-way trade-off) (electronic supplementary material, figures S1b, S3). The paired trade-off between $\hat{r_{\text{2}}}$ and AR (electronic supplementary material, figure S1a) would see the empirical wing forms generally occupying regions of far poorer optimality compared with their position in the paired-trade-offs for which AR was omitted, and especially in the $\hat{r_{\text{2}}}$ and VMS plot in which regions of higher optimality scoring cover a much greater portion of the tested shape space (electronic supplementary material, figure S1c). These observations are indicative of stronger functional constraint in trade-offs with AR included and corroborate previous claims that aspect ratio is the primary morphological trait enabling flight function in insect wings [24,56]. Empirical occupation of the Pareto optimality landscape shows dense clustering of observed taxa is within an area of semi-optimal flight efficiency, neither performing well nor poorly with reference to the three-way trade-off between AR, $\hat{r_{\text{2}}}$ and VMS (figures 3*d* and 5). The empirical wing shapes are not optimized for individual traits, yet there is some evidence for functional optimization as the distribution of empirical wing morphologies show general consistency with the contour of the optimality landscape (figure 5). The theoretical wing forms least optimized for the trade-off are those at lowest PC2 values (minor variation between size of apical tip and humeral base) and those generally broad across the planform, associated with low AR. These forms do not occur in Hymenoptera. The results also show dense occupation of hymenopteran forewings in shape space of moderately high optimality (~0.8), with some overlap at the Pareto front. Our chosen functional metrics evaluate the physical performance of wing shapes and reaffirm Hymenoptera as capable fliers, as well as the relevance of wing shape in examining flight efficiency. However, there are many different survival strategies and flight modes which can compensate for relatively lower functional optimization defined by wing shape metrics. Possible examples include performance measures incalculable from theoretical wing outlines alone (e.g. manoeuvrability), wing beat frequency, as well as additional aspects of body architecture (e.g. flight musculature or venation networks) or proximity and access to energy-rich food sources [14–16,92–94]. While we find some support for functional optimization by natural selection, our results align with previous claims that the morphologies of isolated traits cannot be solely explained by selection towards functional optima. Our findings highlight the applicability and importance of using theoretical morphology and optimality testing, removed from assumptions of functional optimality in nature, in analyses of evolutionary morphology [95,96].

## 5. Conclusion

The disparity of hymenopteran wing morphologies is confined to a subset of the theoretical shape space, with a high level of convergence across the four major hymenopteran clades. The theoretical framework applied in this study has allowed us to reject the commonplace assumption that wing morphologies are optimized for flight performance. Empirical wing forms occupy an area of shape space that is neither at peak optimality for flight efficiency, nor are they poorly optimized. This shows that while there is clearly evidence of functional adaptation in the evolution of hymenopteran wing form, it cannot be considered the sole driver. Wing size and shape covary and there is a significant but weak phylogenetic signal in the evolution of hymenopteran wing morphology, but there is no significant relationship with the environmental factors we considered. It can therefore be concluded that developmental factors and, to a lesser extent, the legacy of evolutionary history on the scope of wing variation act together alongside function in limiting the disparity of hymenopteran forewings.

## Data Availability

The wing outline files, time-calibrated phylogeny, and R code are archived and accessible through Figshare [97]. The Matlab code is available for download from [98].
